# Anomalous Foramina Through the Greater Wing of the Sphenoid Bone: A Radio-Anatomical Study on a Cohort of Dry Skulls from the Interbellum Period Using MDCT

**DOI:** 10.3390/diagnostics16060908

**Published:** 2026-03-19

**Authors:** Andra-Ioana Baloiu, Octavian Munteanu, Andrei Dorian Soficaru, Iuliana-Raluca Gheorghe, Andreea-Nicoleta Marinescu, Ioan-Andrei Petrescu, Răzvan Stănciulescu, Iulian Mirel Slavu, Florin Mihail Filipoiu

**Affiliations:** 1 Doctoral School, “Carol Davila” University of Medicine and Pharmacy, 020021 Bucharest, Romania; andra-ioana.baloiu@drd.umfcd.ro; 2 Department of Anatomy, “Carol Davila” University of Medicine and Pharmacy, 050474 Bucharest, Romania; ioan-andrei.petrescu@drd.umfcd.ro (I.-A.P.); razvan.stanciulescu@umfcd.ro (R.S.); iulian.slavu@umfcd.ro (I.M.S.); florin.filipoiu@umfcd.ro (F.M.F.); 3 “Francisc I. Rainer” Institute of Anthropology, Romanian Academy, 010711 Bucharest, Romania; asoficaru@yahoo.com; 4 Department of Marketing and Medical Technology, Faculty of Medicine, “Carol Davila” University of Medicine and Pharmacy, 050474 Bucharest, Romania; raluca.gheorghe@umfcd.ro; 5 Department of Radiology, “Carol Davila” University of Medicine and Pharmacy, 050474 Bucharest, Romania; 6 Department of Radiology, Emergency University Hospital of Bucharest, 050098 Bucharest, Romania

**Keywords:** dry skull, inconstant foramen, foramen of Vesalius, foramen of Arnold, CT examination

## Abstract

**Background:** The foramen of Arnold (FA) and foramen of Vesalius (FV) are two inconstant small openings through the greater wing of the sphenoid bone. When FA is present, the lesser petrosal nerve passes through it. FV usually contains an emissary vein that connects the cavernous sinus to the pterygoid plexus. **Objectives:** To assess the presence, unilaterally or bilaterally, of the aforementioned inconstant foramina in order to gain a better insight into the anatomic variability of the middle cranial fossa. **Methods:** We analyzed five hundred random unenhanced CT examinations of human dry skulls from the “Francisc I. Rainer” Craniological Collection of the Human Anthropological Institute in Bucharest, Romania. The collection holds substantial anthropological and radiological value, as dry skulls allow the use of higher spatial-resolution imaging parameters and thus better detection of the small osseous structures. All scans were performed on the same Multi-Detector Computed Tomography (MDCT) scanner (Canon Aquilion One 64 slice, Canon Medical Systems Corporation) in the Department of Radiology of the Bucharest Emergency University Hospital. After collecting data, appropriate statistical analysis was performed. **Results:** FA was absent in 355 (71%) skulls and present in 145 (29%) skulls, bilaterally in 33 (6.6%) skulls, and unilaterally in 112 (22.4%) skulls. FV was absent in 151 (30.2%) skulls and present in 349 (69.8%) skulls, bilaterally in 223 (44.6%) skulls, and unilaterally in 126 (25.2%) skulls. **Conclusions:** The great variability in the prevalence of these foramina may be partly explained by the different methods of analysis of the skull base, as well as the different population subgroups on which the research has been focused. Knowledge of this variability holds great importance for anatomists, anthropologists, as well as clinicians who interact with this complex area.

## 1. Introduction

Having a precise and thorough understanding of the intricate anatomical variability is essential for effectively diagnosing and managing various medical conditions. These variations may affect the course of surgical procedures, as well as non-surgical interventions, when addressing a range of medical issues, including infections, genetic disorders, or tumors. Recognizing these differences is paramount for healthcare professionals, as it enables them to tailor treatments to individual patient needs, ultimately leading to improved outcomes and enhanced patient safety.

The sphenoid bone is a critical anatomical structure, forming the largest portion of the middle cranial fossa. It constitutes both the anterior and medial regions of this area’s floor, while also forming a significant part of the posterior wall of the orbit. This complex bone is characterized by its unique three-dimensional shape, which includes a central body along with two lesser wings that extend in an antero-lateral direction. Additionally, it features two larger wings that project outward laterally on either side, enhancing the structural integrity of the cranial cavity. In addition to these features, the sphenoid bone also includes two anteroinferior pterygoid processes, which further contribute to its intricate anatomy and the overall architecture of the skull [1]. In the center, the body of the sphenoid bone houses the sphenoid sinus, superior to which is the sella turcica, with a saddle-shaped depression, known as the hypophyseal fossa or pituitary fossa, where the pituitary gland is found [2]. The greater wings of the sphenoid bone are notable for housing several key foramina that serve as critical passageways for various nerves and blood vessels. Arranged from anteriorly and medially to posteriorly and laterally, the first of these foramina is the foramen rotundum, through which the maxillary division of the trigeminal nerve traverses. This passage is essential for sensory innervation in the mid-facial region. Postero-laterally lies the foramen ovale, an opening that allows for the passage of the accessory meningeal artery, as well as the mandibular division of the trigeminal nerve. Occasionally, the lesser petrosal nerve may also pass through this foramen, contributing to its clinical significance. Lastly, the foramen spinosum is located posteriorly and contains both the middle meningeal artery and vein. Additionally, it houses the meningeal branch of the mandibular division of the trigeminal nerve, which provides important sensory feedback from the meninges. Notably, all of these foramina may also accommodate emissary veins, which help in venous drainage and facilitate communication between the extracranial and intracranial venous systems [3].

In addition to the well-established anatomical structures that also may exhibit variations, our research primarily focused on two inconstant foramina: the foramen of Vesalius, which is also referred to as the foramen venosum or the emissary sphenoidal foramen (FV), and the foramen of Arnold, commonly known as the foramen petrosum or canaliculus innominatus (FA). When present, the FA is a small but significant opening located within the greater wing of the sphenoid bone, most frequently posterior to the foramen spinosum and medial to both the foramen ovale and foramen spinosum [2,3]. This foramen serves an important function as it houses the lesser petrosal nerve, which is composed of fibers that branch from the glossopharyngeal and facial cranial nerves [4]. FV also inconstantly presents itself in the greater sphenoidal wing, anteromedially to the foramen ovale and lateral to the foramen rotundum [5]. It is noteworthy for its role in venous drainage and communication within the cranial cavity, as it usually contains an emissary vein that connects the cavernous sinus to the pterygoid plexus; therefore, it is a possible pathway via which extra-cranial infections can enter the middle fossa [6].

The aim of this paper is to assess the presence, unilaterally or bilaterally, of the aforementioned inconstant foramina on the greater wing of the sphenoid bone, to gain a better insight into the anatomic variability of the middle cranial fossa, with great importance for anatomists, anthropologists, as well medical professionals, particularly in fields such as neurology and surgery, where precise knowledge of anatomical pathways is imperative for effective diagnosis and treatment.

## 2. Materials and Methods

### 2.1. Specimen Selection

**Figure 1:**
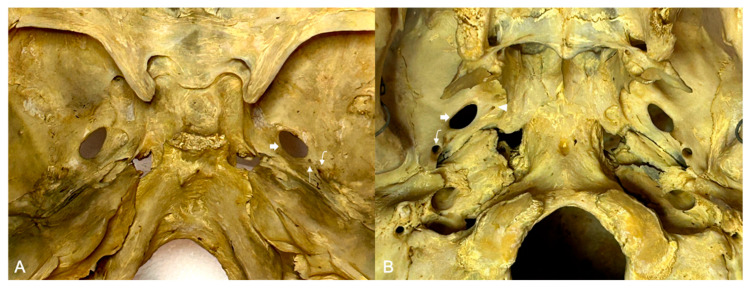
Photographs of dry skulls included in the study. (**A**) Superior view of the central part of the middle cranial fossa. Note a small FA (thin arrow) present on the right side, between foramen ovale (thick arrow) and foramen spinosum (curved arrow). (**B**) Inferior view of the skull base. FV (arrowhead) is present on the right side, anteromedially to foramen ovale (thick arrow); the curved arrow indicates foramen spinosum.

We conducted a comprehensive analysis of 500 random unenhanced computed tomography (CT) examinations of human dry skulls from the “Francisc I. Rainer” Craniological Collection, which is housed at the Institute of Anthropology in Bucharest, Romania. To ensure the integrity and objectivity of our research, we employed a systematic randomization process, which involved selecting a subset of skulls from a digitalized inventory list using a random number generator application. All the skulls had been identified (216 females, 284 males, aged 20–82 years) and are of Romanian nationality, dating back to the interwar period (Figure 1). This retrospective study adhered to all relevant principles of the Declaration of Helsinki. The Ethics Committee of the “Carol Davila” University of Medicine and Pharmacy in Bucharest approved the digitalization of the Craniological Collection (approval no. 14355; 30 May 2024). The Ethics Committee of the “Francisc I. Rainer” Institute of Anthropology approved the digitalization of the Craniological Collection (approval no. 847; 29 May 2024). The Ethics Committee of the Emergency University Hospital of Bucharest approved the digitalization of the Craniological Collection (approval no. 32986; 27 May 2024). Exclusion criteria included any CT scans that were deemed technically suboptimal, severe traumatic or destructive lesions that impaired the evaluation, and absence of the greater wing of the sphenoid unilaterally.

We analyzed the presence of the two inconstant foramina on the greater wing of the sphenoid bone: FA and FV, determining the variability (whether each of the foramina were absent, present unilaterally or bilaterally) by age and sex.

### 2.2. Imaging

All computed tomography (CT) scans were conducted using a Multi-Detector Computed Tomography (MDCT) scanner, specifically the Canon Aquilion One 64 slice model, manufactured by Canon Medical Systems Corporation (Otawara, Tochigi, Japan). The scans were performed at the Department of Radiology within the Emergency University Hospital of Bucharest. To ensure the highest quality of imaging, we performed multiple CT acquisitions, each with varying imaging parameters. This process aimed to identify the optimal settings necessary for producing clear and accurate images, enhancing the reliability of our imaging and facilitating a thorough analysis of the anatomical features under investigation. The final imaging parameters (tube voltage of 120 kWp, X-ray tube current of 19 mA) were approved by three researchers (A.I.B., A.N.M. and R.S.) ensuring optimal image contrast and signal-to-noise ratio for visualization of the small foramina. Once the imaging parameters were established, we performed several other CT acquisitions in order to determine which device is best suited for supporting the skulls in anatomical position, without compromising the three-dimensional reconstructions. We tested cardboard boxes, various foam head supports compatible with our CT scanner, radiolucent positioning sponges, and polystyrene foam circular rings. We chose to position the skulls by resting the occipital bone on a polystyrene foam circular ring with an internal diameter of 15 cm, which has the lowest density of approximately −970 Hounsfield Units (HUs), while the air surrounding the skulls has a density of −990 to −1000 HUs. Periodic air calibrations were performed at manufacturer-specified intervals to maintain the accuracy and stability of the HU scale and to correct for potential detector drift in the CT system. We chose not to scan the mandibles, to provide an unobstructed view of the outer skull base. The CT scanner table height was adjusted for every skull to ensure the correct position in the isocenter of the gantry. The CT three-laser system equipped with line lasers in the three anatomical planes (axial, sagittal, coronal) aided the symmetrical positioning. Each skull was positioned by aligning the axial laser with the superior margin of both orbits, the sagittal laser with the internasal suture, intermaxillary suture and the midline of the foramen magnum, and the coronal laser with both external acoustic meatus.

**Figure 2:**
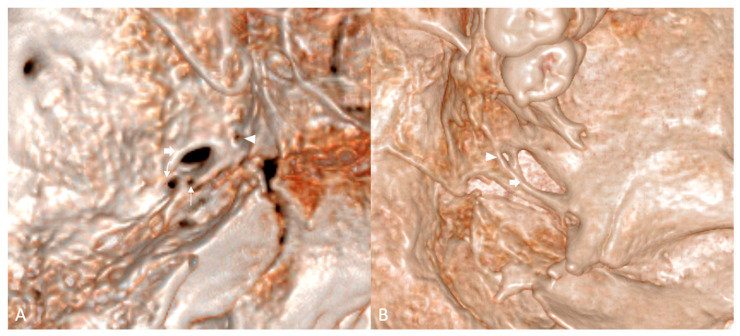
Three-dimensional CT reconstructions of dry skulls included in the study. (**A**) Left middle cranial fossa; note the FA (thin arrow) present anteromedially to foramen spinosum (curved arrow), and the FV (arrowhead) anteromedially to foramen ovale (thick arrow). (**B**) Inferior view of the left greater wing of the sphenoid bone; note the FV (arrowhead) anteromedially to foramen ovale (thick arrow).

Multiplanar bidimensional reconstructions were performed in the three anatomical planes (axial, coronal and sagittal) using the bone window, with a slice thickness of 0.5 mm. Three-dimensional reconstructions of each skull were also performed for better assessment of the skull base (Figure 2). All scans were evaluated by the same researcher (A.I.B.) using a dedicated workstation.

### 2.3. Examiner Consistency

One hundred randomly selected images were observed again by the same investigator one month after the first round of observations. Examiner consistency was assessed for all variables. Variables were highly similar between the first and second rounds of observations, with correlation coefficients of 0.85–0.96.

### 2.4. Statistical Analyses

The collected data was processed and analyzed with the help of frequency and percentage in Statistical Package for the Social Sciences (ver.20; IBM Corporation, Armonk, NY, USA).

## 3. Results

A total of 500 CT examinations of dry skulls (216 females, 284 males) with ages ranging between 20 and 82 years (mean age 46.73) were included in the present study.

### 3.1. Foramen of Arnold

**Figure 3:**
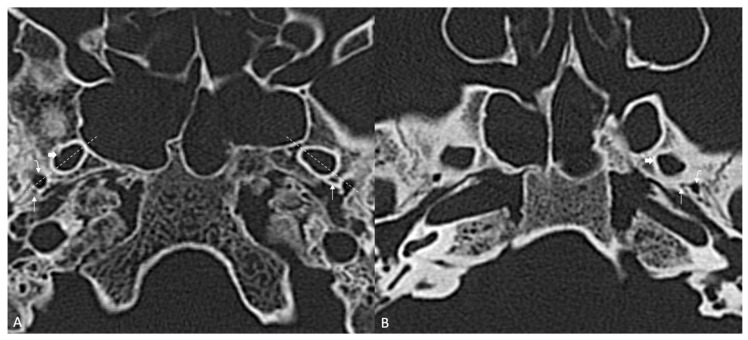
Axial sections through the sphenoid sinus and greater wings of the sphenoid bone, bone window. (**A**) FA (thin arrow) is present bilaterally. Note the dotted line passing through the long axis of foramen ovale (thick arrow) and extending to foramen spinosum (curved arrow), from anteriorly and medially to posteriorly and laterally. On the right side, the FA is located postero-medially to foramen spinosum, while on the left side it is situated anteromedially. (**B**) FA (thin arrow) is present unilaterally on the left side, between the foramen ovale (thick arrow) and foramen spinosum (curved arrow).

We considered FA present only in skulls that presented a well-defined foramen medially to a line passing through the long axis of foramen ovale and extending to foramen spinosum. We identified FA either anteromedially or postero-medially to foramen spinosum (Figure 3). The foramen was present in 145 (29%) skulls and absent in 355 (71%) skulls. Among the skulls in which this foramen existed, it was present bilaterally in 33 (6.6%) skulls, and unilaterally in 112 (22.4%) skulls (64 (12.8%) skulls on the left side, 48 (9.6%) skulls on the right side). In the female subgroup (*n* = 216), FA was absent in 164 (75.9%) skulls, and present in 52 (24.1%) skulls. We detected FA in 12 (5.6%) skulls bilaterally, and unilaterally in 40 (18.5%) skulls (23 (10.6%) skulls on the left side, 17 (7.9%) skulls on the right side). In the male subgroup (*n* = 284), FA was absent in 191 (67.2%) skulls, and present in 93 (32.8%) skulls. We detected FA in 41 (14.4%) skulls bilaterally, and unilaterally in 52 (25.3%) skulls (41 (14.4%) skulls on the left side, 31 (10.9%) skulls on the right side). The results are summarized in Table 1.

### 3.2. Foramen of Vesalius

**Figure 4:**
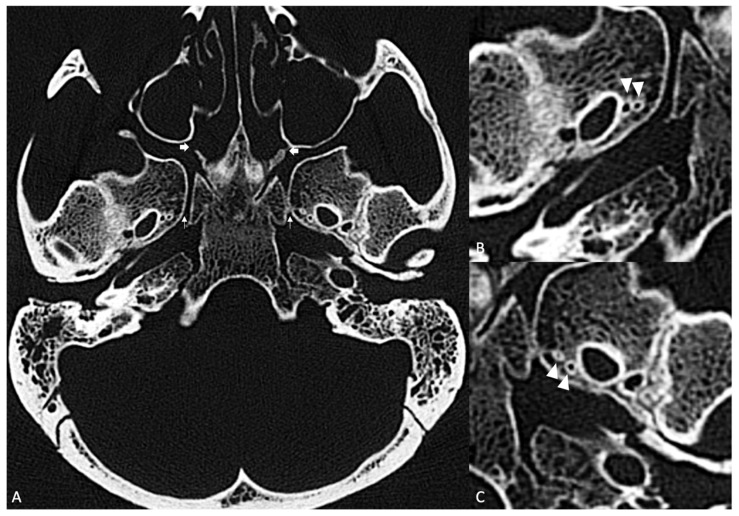
(**A**) Axial section through the greater wings of the sphenoid bone bilaterally in a skull presenting with bilateral duplicated FV, bone window; two of the passageways to and from the pterygopalatine fossae; the vidian canals (thin arrows) and sphenopalatine foramina (thick arrows) are also noted. (**B**) Detailed image centered on the right middle cranial fossa. Note the two FVs (arrowheads) on the right side. (**C**) Detailed image centered on the left middle cranial fossa. Note the two FVs (arrowheads) on the left side.

FV was detected anteromedially to foramen ovale in all specimens. We detected no confluence or assimilation with foramen ovale; however, 12 (2.4%) skulls presented with double FV, unilaterally in 9 (1.8%) skulls and bilaterally in 3 (0.6%) skulls (Figure 4). FV was present in 349 (69.8%) skulls and absent in 151 (30.2%) skulls. Among the skulls in which this foramen existed, it was present bilaterally in 223 (44.6%) skulls, and unilaterally in 126 (25.2%) skulls (70 (14%) skulls on the left side, 56 (11.2%) skulls on the right side). In the female subgroup (*n* = 216), FV was absent in 73 (33.8%) skulls, and present in 143 (66.2%) skulls. We detected FV in 97 (44.9%) skulls bilaterally, and unilaterally in 46 (21.3%) skulls (28 (13%) skulls on the left side, 18 (8.3%) skulls on the right side). In the male subgroup (*n* = 284), FA was absent in 78 (27.5%) skulls, and present in 206 (72.5%) skulls. We detected FV in 126 (44.4%) skulls bilaterally, and unilaterally in 80 (28.1%) skulls (42 (14.7%) skulls on the left side, 38 (13.4%) skulls on the right side). The results are summarized in Table 2.

## 4. Discussion

In this study, we detected FA to be present in 29% (*n* = 145) of skulls, 6.6% (*n* = 33) of which bilaterally. A similar radiological study using High-Resolution unenhanced CT scans of 123 patients performed by Ginsberg et al. [7] detected a prevalence of 16%, with 0.81% (*n* = 1) of patients presenting the foramen bilaterally. We did not detect any duplicated foramina in the present study. A study on Cone-Beam Computed Tomography (CBCT) examinations in a Turkish population group observed the presence of FA in only 17.1% (*n* = 60) [8]. Another radiological study, based on 300 CT examinations in an Italian population group [9], detected the presence of the foramen in 14% (*n* = 42), with a prevalence of 14.7% in males and 13.3% in females, although, in their study, the FA was defined as a “small opening connecting the foramen spinosum and ovale”. In the present study, we detected a higher prevalence of 32.8% in males and 24.1% in females. Kumar et al. [10] analyzed 50 adult human dry skulls and determined that FA was found only unilaterally in five (10%) skulls.

To our knowledge, the only study that discovered a higher prevalence (percentage-wise) of FA performed on human dry skulls is the study of Sankaran et al. [11], performed on 64 dry skulls of unknown sex and Indian origin, with 27 (42.18%) skulls with bilateral FA and 4 (6.25%) skulls with unilateral foramen.

Although being considered a rather “obscure anatomical structure” [7], and taking into account the scarcity of scientific research on the matter, proper knowledge of the variability and prevalence of FA reduces the chances of confusion between the greater and lesser petrosal nerves, thus impacting the outcome of certain middle cranial fossa interventions [12].

We determined that FV was present in 69.8% (*n* = 349) of the evaluated skulls, bilaterally in 44.6% (*n* = 223) of the skulls. To the best of our knowledge, this is one of the highest prevalences detected so far in the literature. There is great variability in the results. Similar high values have been determined in several other radiological studies analyzing CT examinations (either CBCT or MDCT) of patients: 67.7% (*n* = 338) in a Serbian population group [13], 73.1% (*n* = 190) in a Turkish population group [14], and as high as 79.5% (*n* = 98) in an American population group [7], although one CBCT study determined a lower prevalence of 28.1% (*n* = 89) in a Turkish population group [15]. Other morphological studies on dry skulls have determined a variable prevalence of this foramen; for example, a study performed on an Indian population group determined a total prevalence of 60% (*n* = 90), 32% (*n* = 29) bilaterally and 35% (*n* = 32) unilaterally [16]. Conversely, a study performed on 400 macerated dry skulls [17] determined the prevalence of FV to be 33.75% (*n* = 135), bilaterally in 15.5% (*n* = 62).

A few authors, such as Kodama et al. [18], detected the FV to be more frequent bilaterally. This has also been the case in our study, with a bilateral prevalence of 44.6% (*n* = 223) and a unilateral prevalence of 25.2% (*n* = 126). We encountered no significant difference between the two sides, and no significant differences between the two genders, although Chaisuksunt et al. [6], in their study on 377 dry skulls, encountered the foramen more frequently on the left side, and in male subjects. Furthermore, Lanzieri et al. [5] determined this variant structure to be more frequent than commonly thought, and rather symmetrical, when present bilaterally, suggesting that asymmetry could be associated with pathological changes. Moreover, several studies describe intracranial hemorrhages following injury of the emissary veins passing through FV reported after a trigeminal nerve block for mandibular nerve pathology [17,19].

The vascular structures and cranial nerves develop before the base of the skull [20]; therefore, the formation of accessory foramina on the greater wing of the sphenoid bone is dependent on the neural and vascular disposition of each individual. Nonetheless, we consider that a good understanding of the regional anatomy and its variations plays a crucial role in the success of various interventions performed in this delicate region.

Apart from the substantial anthropological value of the collection, dry skulls are preferable to the intact human head in radiological studies of osseous structures, as the absence of soft tissues increases bone–background contrast, reduces beam-hardening artifacts and allows the use of higher spatial-resolution imaging parameters.

The limitations of the present study include: (1) the 500 CT examinations pertain to crania of individuals of Romanian nationality; therefore, the evidence brought here should be further verified in other population groups; (2) the crania included in the study are approximately a century old, having sustained a certain degree of deterioration; (3) although the acquisition parameters have been adapted to obtain high-resolution images, the small diameter of these foramina hinders their detection in some individuals; (4) the data were collected through evaluation performed by a single experienced reader; however, assessment by at least two independent readers would have been preferable to reduce observer-related variability.

We are collecting data for a future study involving contemporary patient cerebral CT examinations to compare the frequencies of these two accessory foramina.

## 5. Conclusions

FA and FV are two inconsistent foramina located on the greater wing of the sphenoid bone. In this study, we detected FA to be present in 29% of the skulls, in 6.6% of which bilaterally, and unilaterally in 112 (22.4%) skulls (64 (12.8%) skulls on the left side, 48 (9.6%) skulls on the right side). FV was present in 69.8% of the evaluated skulls, bilaterally in 44.6%, and unilaterally in 126 (25.2%) skulls (70 (14%) skulls on the left side, 56 (11.2%) skulls on the right side). The great variability in the prevalence of these foramina may be partly explained by the different methods of analysis of the skull base, as well as the different population subgroups on which the research has been focused. To our knowledge, this is the first study that analyzes 500 dry skulls pertaining to identified individuals from the interwar period. Therefore, this study contributes to understanding the anthropological and anatomical variability of this foramen located in the greater wing of the sphenoid bone.

## Figures and Tables

**Table 1:** Variability of foramen of Arnold by side and sex.

	Frequency	Percent	Female Frequency (%)	Male Frequency (%)
Absent	355	71.0	164 (75.9)	191 ( 67.3)
Left	64	12.8	23 (10.6)	41 (14.4)
Right	48	9.6	17 (7.9)	31 (10.9)
Bilateral	33	6.6	12 (5.6)	21 (7.4)
Total	500	100.0	216 (100)	284 (100)

**Table 2:** Variability of foramen of Vesalius by side and sex.

	Frequency	Percent	Female Frequency (%)	Male Frequency (%)
Absent	151	30.2	73 (33.8)	78 (27.5)
Left	70	14.0	28 (13)	42 (14.7)
Right	56	11.2	18 (8.3)	38 (13.4)
Bilateral	223	44.6	97 (44.9)	126 (44.4)
Total	500	100.0	216 (100)	284 (100)

## Data Availability

The original contributions presented in this study are included in the article. Further inquiries can be directed to the corresponding author.
